# Fungal Keratitis Caused by *Talaromyces coalescens*: A Case Report

**DOI:** 10.1007/s11046-023-00738-w

**Published:** 2023-05-09

**Authors:** Daisuke Todokoro, Tomoko Miyakubo, Koichi Makimura, Takashi Tamura, Aya Komori, Hideo Akiyama

**Affiliations:** 1 Department of Ophthalmology, Gunma University Graduate School of Medicine, 3-39-15 Showa-machi, Maebashi-city, Gunma 371-8511 Japan; 2 Teikyo University Institute of Medical Mycology, 2-11-1 Koga, Itabashi-ku, Tokyo, 173-8605 Japan

**Keywords:** *Talaromyces coalescens*, Fungal keratitis, Voriconazole, Natamycin

## Abstract

Fungal keratitis is a severe corneal infection, and the causative fungi include various rare fungal species. Fungal keratitis caused by *Talaromyces* species has yet to be reported, and there is no information about this fungus as a cause of keratitis. A 77-year-old man developed fungal keratitis while waiting for a donor cornea due to bullous keratopathy in his left eye. Fungal culture of a corneal scraping grew filamentous fungi, which were morphologically identified as *Paecilomyces* species. The corneal infection did not improve after topical administration of 1% voriconazole, and ribosomal DNA sequencing definitively verified the fungus to be *Talaromyces coalescens*. The lesion gradually improved after switching to topical 5% natamycin. Antifungal susceptibility tests determined the high minimum inhibitory concentrations of voriconazole to be > 8 μg/mL. This is the first report of *Talaromyces* fungal keratitis. Clinicians, especially those in ophthalmology, need to be aware of this rare fungus.

## Introduction

Fungal keratitis is a severe corneal infection, with more than one million people affected per year worldwide [1]. In Japan, fungal keratitis accounts for 6.3% of the total for infectious keratitis [2], with the causative fungi including species of *Candida* (43.6%), *Fusarium* (24.5%), *Alternaria* (6.4%), and *Aspergillus* (3.2%) among various other fungal species [3]. However, fungal keratitis caused by *Talaromyces* species has not been reported to date, and there is yet to be any information regarding the clinical findings, fungal species, antifungal susceptibility, effective treatment, and visual outcomes. In this report, we describe the first case of *Talaromyces coalescence* fungal keratitis.

## Case Report

**Fig. 1:**
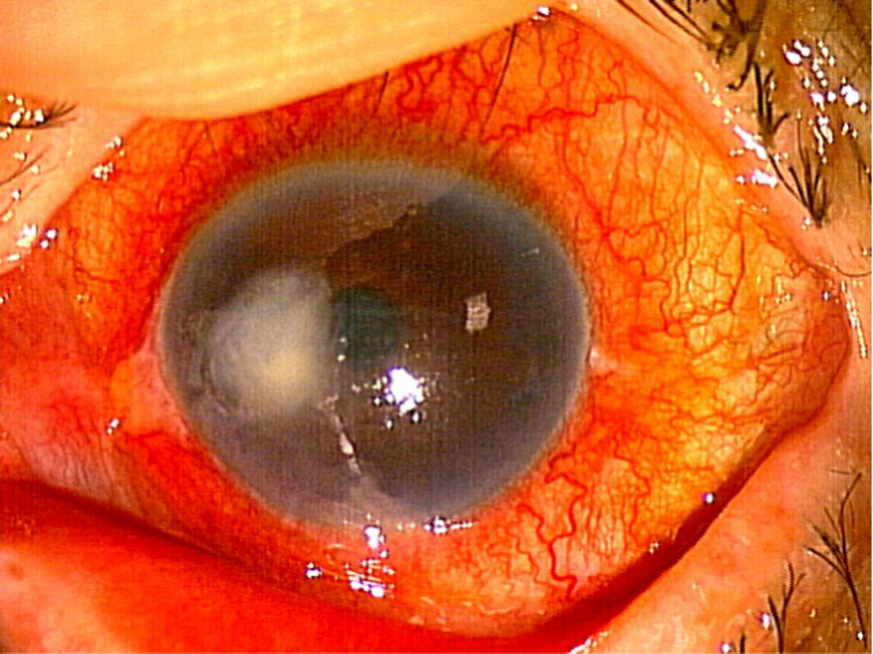
Slit-lamp findings for the left eye at the onset of keratitis showed corneal ulcer accompanying feathery infiltration in the temporal mid-peripheral cornea. Ciliary injection and hypopyon were also observed

**Fig. 2:**
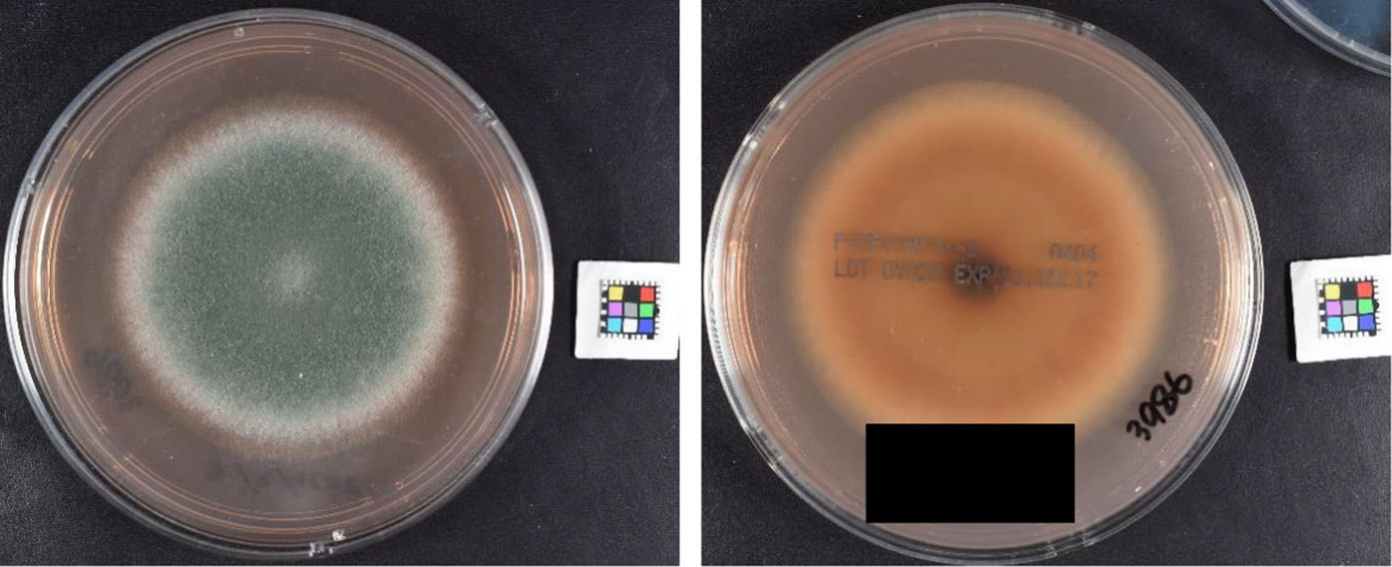
A giant colony grown on a potato-dextrose agar plate grown at 28 °C was found to be floccose and greenish on the surface side (left) and orange-brown on the reverse side (right). Red soluble pigment exuded into the medium

**Fig. 3:**
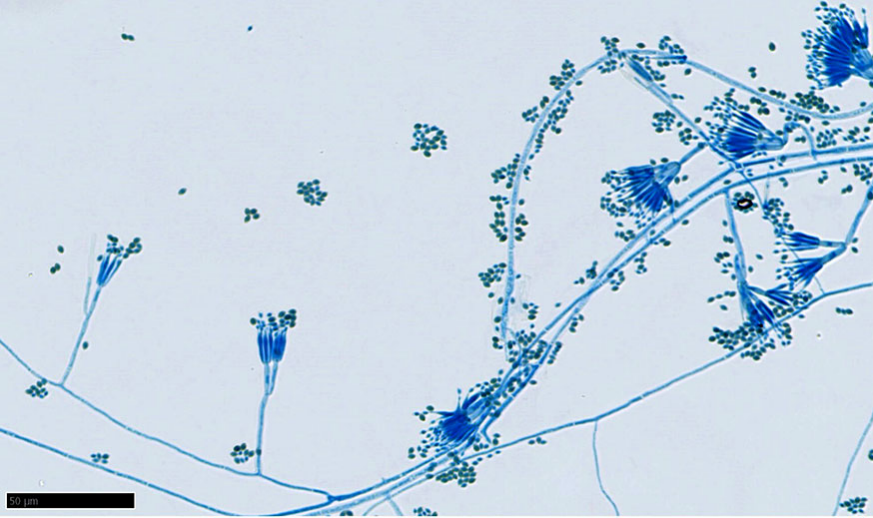
Slide culture prepared with lactophenol cotton blue showed chains of single cell conidia born on phialides with a brush-like appearance (× 400)

A 77-year-old man with a history of hypertension, diabetes mellitus, and cervical spondylosis was being treated for glaucoma by a local ophthalmologist. Due to the development of corneal edema accompanying keratoprecipitates in his left eye, the ophthalmologist began a 5-month treatment with topical 0.1% betamethasone and 3% acyclovir ointment. However, due to the lack of a response, he was referred to our department. Best-corrected visual acuity at his initial visit to our department was 1.0 and 0.4 in the right and left eye, respectively. Intraocular pressure was 11 mmHg in both eyes. Corneal endothelial cell density in the right eye was found to have decreased to 600 cells/mm^2^ and was unmeasurable in the left eye due to corneal edema. Using aqueous humor, a multiplex PCR test did not detect any DNA of herpes simplex virus, varicella-zoster virus, or cytomegalovirus. We diagnosed his left eye as bullous keratopathy due to an unknown cause, and placed the patient on a corneal donor waiting list for a corneal transplantation. Topical steroids were continued in order to suppress relapse of the intraocular inflammation. However, after 4 months of waiting for a donor cornea, he developed ciliary injection and discomfort of his left eye. Ophthalmological examination showed inflammation in the left anterior chamber, corneal ulcer, feathery corneal infiltration, endothelial plaque, and hypopyon (Fig. 1). As clinical findings suggested fungal keratitis, we performed corneal scraping for diagnosis. However, direct microscopy did not find any fungal elements. Thus, we started a combination of topical antibiotics, 1.5% levofloxacin, and 0.5% cefmenoxime 6 times a day, respectively, with discontinuation of the topical betamethasone. Our department’s clinical laboratory examined a 2-week-old fungal culture that grew filamentous fungi and identified *Paecilomyces* species from the slide culture image. As a result, we started topical 1% voriconazole hourly. As the corneal infection did not improve after a further 3 weeks, we performed ribosomal DNA sequencing of the internal transcribed spacer (ITS), with results demonstrating that the fungus was not *Paecilomyces* species but rather *Talaromyces* species. Therefore, the patient was switched from topical voriconazole to topical 5% natamycin. After a 2-month natamycin treatment, there was gradual improvement of the corneal ulcer with some scarring after the treatment. He finally underwent corneal transplantation, with the best-corrected visual acuity improving to 1.2. The isolated fungus was ultimately identified as *T. coalescens* from both the ITS and beta tubulin gene (BT1) sequences. The DNA sequences (ITS and BT1) were deposited in the DDBJ database (LC744196 and LC744197, respectively). An antifungal susceptibility test was performed based on the CLSI M38-A2 standard. The minimum inhibitory concentrations (MICs) of the strain were as follows: amphotericin B, 1 µg/mL; natamycin, 2 µg/mL; fluconazole, > 64 µg/mL; miconazole, > 16 µg/mL; and voriconazole, > 8 µg/mL, respectively (Figs. 2, 3).

## Discussion

*Talaromyces* species are widely distributed in environments, being primarily found in the soil. This genus was formerly classified as the *Penicillium* species in the subgenera *Biverticillium* [4]. The slide culture images of the *Talaromyces* species are similar to those of the *Penicillium* species and also *Paecilomyces lilacinus* (current name, *Purpureocillium lilacinum*), which is one of the major causes of fungal keratitis. In the present case, the isolated fungal strain was initially misidentified as *Paecilomyces* or *Purpureocillium* species. As most strains of *Purpureocillium lilacinum* are susceptible to voriconazole, we empirically treated this case with topical voriconazole [5, 6]. However, the corneal infection did not respond to the topical voriconazole. Thus, it was not until later that molecular identification finally revealed that the causative fungus was actually *T. coalescens*. An antifungal susceptibility test indicated that this strain was not susceptible to voriconazole, which was consistent with the clinical course. Human infections caused by *T. coalescens*, which include ocular infection, have not been reported to date. Thus, this case is the first report of *T. coalescens* fungal keratitis. Clinicians, especially those in ophthalmology, need to be aware of this rare fungus.

## References

[1] Brown L, Leck AK, Gichangi M, Burton MJ, Denning DW (2021). The global incidence and diagnosis of fungal keratitis. Lancet Infect Dis.

[2] Khor WB, Prajna VN, Garg P, Mehta JS, Xie L, Liu Z, Padilla MDB, Joo CK, Inoue Y, Goseyarakwong P, Hu FR, Nishida K, Kinoshita S, Puangsricharern V, Tan AL, Beuerman R, Young A, Sharma N, Haaland B, Mah FS, Tu EY, Stapleton FJ, Abbott RL, Tan DT (2018). The Asia Cornea Society Infectious Keratitis Study: a prospective multicenter study of infectious Keratitis in Asia. Am J Ophthalmol.

[3] Inoue Y, Ohashi Y, Shimomura Y, Sotozono C, Hatano H, Fukuda M, Eguchi H, Araki-Sasaki K, Suzuki T, Hoshi S, Asari S, Sunada A, Kimura K, Yaguchi T, Makimura K (2022). Multicenter prospective observational study of fungal keratitis in Japan: analyses of culture-positive cases. Jpn J Ophthalmol.

[4] Samson RA, Yilmaz N, Houbraken J, Spierenburg H, Seifert KA, Peterson SW, Varga J, Frisvad JC (2011). Phylogeny and nomenclature of the genus *Talaromyce*s and taxa accommodated in *Penicillium* subgenus *Biverticillium*. Stud Mycol.

[5] Chew R, Dorman A, Woods ML (2016). *Purpureocillium lilacinum* keratitis: a case series and review of the literature. Can J Ophthalmol.

[6] Todokoro D, Yamada N, Fukuchi M, Kishi S (2014). Topical voriconazole therapy of *Purpureocillium lilacinum* keratitis that occurred in disposable soft contact lens wearers. Int Ophthalmol.

